# Acquired Resistance to Antituberculosis Drugs

**DOI:** 10.3201/eid2411.180465

**Published:** 2018-11

**Authors:** Htin Lin Aung, Wint Wint Nyunt, Yang Fong, Bruce Russell, Gregory M. Cook, Si Thu Aung

**Affiliations:** University of Otago, Dunedin, New Zealand (H.L. Aung, B. Russell, G.M. Cook);; Ministry of Health and Sports, Yangon, Myanmar (W.W. Nyunt);; Massey University, Palmerston North, New Zealand (Y. Fong);; Ministry of Health and Sports, Naypyitaw, Myanmar (S.T. Aung)

**Keywords:** Food safety, drug-resistant, Myanmar, tuberculosis, tuberculosis and other mycobacteria, whole-genome sequencing, antimicrobial resistance

**To the Editor:** We read with great interest the article by Loutet et al. on acquired resistance to antituberculosis drugs in low-burden settings, such as England, Wales, and Northern Ireland (*1*), and support their assertion that detecting acquired resistance should be a priority in high-burden settings. This objective is particularly urgent in Myanmar, where tuberculosis (TB) is highly endemic (*2*) and drug-resistant TB is present through both acquired drug resistance and direct transmission. Unfortunately, the overwhelming number of TB cases precluded routine phenotypic drug susceptibility testing (DST) of first- or second-line drugs, so we began using whole-genome sequencing (WGS), which enabled us to more rapidly diagnose drug-resistant TB (*3*). Here, we briefly describe 2 cases of acquired antituberculosis drug resistance detected by WGS. 

Patient A, diagnosed with rifampin-susceptible TB by Xpert (Cepheid Inc., Sunnyvale, CA, USA), received a treatment regimen containing first-line drugs but failed to achieve smear conversion at the 3-month follow-up. WGS indicated that the isolate was resistant to isoniazid, streptomycin, and rifampin. WGS and phenotypic DST of the isolate at baseline revealed it was resistant to isoniazid and streptomycin. Isolates from before and after treatment differed by 2 single-nucleotide polymorphisms, suggesting that rifampin resistance was acquired during therapy (*4*). Patient B was diagnosed with rifampin-resistant TB and reported that he had started multidrug resistant (MDR) TB treatment 6 months earlier but failed to continue the treatment. WGS and phenotypic DST showed the case had been MDR TB (resistant to isoniazid, rifampin, and streptomycin, but sensitive to amikacin) at baseline but had become pre–extensively drug resistant (amikacin resistance was acquired during treatment). 

Loutet et al. showed that WGS provides an effective way to evaluate TB drug resistance in low-endemicity settings (*5*). We believe WGS is even more vital to help direct MDR TB treatment in high-burden settings, to halt the continued spread of TB. 
